# Robust Measurement-Driven Cardinality Balance Multi-Target Multi-Bernoulli Filter

**DOI:** 10.3390/s21175717

**Published:** 2021-08-25

**Authors:** Biao Yang, Shengqi Zhu, Xiongpeng He, Kun Yu, Jingjing Zhu

**Affiliations:** National Laboratory of Radar Signal Processing, Xidian University, Xi’an 710071, China; bbyang@stu.xidian.edu.cn (B.Y.); xphe@xidian.edu.cn (X.H.); yukun@stu.xidian.edu.cn (K.Y.); jjzhu_2@stu.xidian.edu.cn (J.Z.)

**Keywords:** multiple targets tracking, random finite set, cardinality balance multiple targets multi-Bernoulli filter, correlation function

## Abstract

The multi-target tracking filter under the Bayesian framework has strict requirements on the prior information of the target, such as detection probability density, clutter density, and target initial position information. This paper proposes a novel robust measurement-driven cardinality balance multi-target multi-Bernoulli filter (RMD-CBMeMBer) for solving the multiple targets tracking problem when the detection probability density is unknown, the background clutter density is unknown, and the target’s prior position information is lacking. In RMD-CBMeMBer filtering, the target state is first extended, so that the extended target state includes detection probability, kernel state, and indicators of target and clutter. Secondly, the detection probability is modeled as a Beta distribution, and the clutter is modeled as a clutter generator that is independent of each other and obeys the Poisson distribution. Then, the detection probability, kernel state, and clutter density are jointly estimated through filtering. In addition, the correlation function (CF) is proposed for creating new Bernoulli component (BC) by using the measurement information at the previous moment. Numerical experiments have verified that the RMD-CBMeMBer filter can solve the multi-target tracking problem under the condition of unknown target detection probability, unknown background clutter density and inadequate prior position information of the target. It can effectively estimate the target detection probability and the clutter density.

## 1. Introduction

Multi-target tracking (MTT) is a key technology in both military [1] and civilian [2] fields. MTT is mainly dedicated to the multi-target tracking problem with an unknown and time-varying number in a complex environment. Generally, not all targets can be detected by the sensor, and the sensor will also generate a series of independent and indeterminate amount of clutter measurements. The core idea of MTT is to deal with the correlation between these complex measurements and the target state. The early processing method is to divide the multi-target into multiple single targets (which are associated with all the measurements) and then obtain the joint estimation result of the target state and number, such as joint probabilistic data association [3] (JPDA) filter and multiple hypothesis tracking [4] (MHT) filter. Unfortunately, MTT filters based on data association has greater algorithm complexity due to their combined characteristics, and the tracking performance is severely limited by the number of targets and hardware issues.

Different from the JPDA and MHT algorithms, the random finite set [5] (RFS) theory can avoid the complex data association problem in the multi-target tracking process. In [6], RFS and traditional data association algorithms are compared in detail. The Bayes filter under the RFS framework models the multi-target state and the measurement containing clutter into two RFSs, and the random finite set statistics (FISST) theory [6,7] is employed to solve the uncertainty of the correlation between the multi-target state and measurement in a clutter environment.

Since RFS was proposed, most studies have assumed the background clutter density and target detection probability as priori known information. These studies can be divided into two categories based on whether the track information can be output: The first type is unlabeled filters and the most typical one is probability hypothesis density (PHD) filter [7], which mainly realizes the estimation of multi-target states by passing first-order moments of the multi-target posterior probability density. Subsequently, [8] proposed a cardinality balance probability hypothesis density (CPHD) filter based on the PHD filter by introducing the cardinality distribution, thereby solving the problem of the PHD filter in the estimation of the number of targets. Different from PHD and CPHD filters, the multi-target multi-Bernoulli filter [5] (MeMBer) and the cardinality balance multi-Bernoulli filter [9] (CBMeMBer) based on MeMBer filter characterize the target state as Bernoulli RFS, and achieve the estimation of the multi-target state by transmitting the posterior probability density of the Bernoulli component (BC). MeMBer and CBMeMBer filters do not require clustering when extracting the target state, so their performance will not be affected by the clustering algorithm. Because of the superior performance of MeMBer and CBMeMBer filters, they are widely used in fields such as multi-sensor multi-target tracking [10,11] and video target tracking [12].

The second category is labeled filters, which can output the track of each target, mainly including labeled multiple targets multi-Bernoulli filter (LMB) [13] and δ-generalized labeled multiple targets multi-Bernoulli (δ-GLMB) filter [14]. In recent years, the filter based on RFS theory has attracted extensive attention of scholars due to its superior performance [15]. It has been widely used in radar tracking [16], video tracking [17], cell tracking [18], and other fields.

However, in the above-mentioned MTT filters, it is usually assumed that the background clutter density and the detection probability of the target are prior known knowledge. In fact, the detection probability of target and the background clutter density in the target detection process are usually affected by factors, such as the environment and the distance between the target and radar, so it is difficult to obtain them in advance. Therefore, MTT technology with unknown target detection probability and background clutter density is a major challenge in the MTT problem. The earliest research based on this problem is [19]. The foreground and background modeling techniques are utilized to extract the target detection probability and background clutter density, but its performance depends heavily on the choice of detection method. In addition, the target detection probability and clutter density in [19] are not time-varying. The earliest model that can estimate the time-varying clutter density and the target detection probability is given in [20,21]. The main conception is to model the detection probability density of the target as a Beta distribution, and model clutter as an independent clutter generator that obeys the Poisson distribution. Ref. [20] proposed a robust CPHD/PHD (R-CPHD/PHD) filter that can adaptively learn the clutter density and target detection probability in a linear Gaussian environment. At present, R-CPHD filter has been applied to cells tracking [22]. Refs. [21,23] extended [20] to non-linear problems and cardinality balance multi-Bernoulli (R-CBMeMBer) filter, respectively. Different from the method in [20], Ref. [24] is based on the Poisson multi-Bernoulli mixture (PMBM) filter, modeling the clutter rate as a Gamma distribution. Furthermore, the joint estimation of the unknown clutter density and multi-target state is realized, but the detection probability of the target in [24] is assumed to be known. In [25], the author proposed a PHD/CPHD filter based on inverse Gamma Gaussian mixing, which can dynamically estimate the detection probability of the target, but the author did not consider the unknown clutter density. Ref. [26] modeled the target detection probability as a Beta distribution, and derived in detail the Gaussian closed solution of PMBM filter with unknown detection probability. Additionally, the author did not consider the unknown clutter density. In [27], the ideas in [20,21] are extended to GLMB filter and applied to video target tracking.

In all the MTT filters mentioned above, the new target is modeled as a distribution with a fixed mean and covariance, and then the posterior probability density of this distribution is predicted and updated to realize the estimation of the multi-target state. This method is awkward. If one wants to achieve the desired tracking effect, he not only needs to know the prior information of the initial position of the target, but also needs to initialize a larger covariance matrix [23]. However, in the sequential Monte Carlo (SMC) implementation, a larger covariance matrix means that more particles are needed to capture the target, estimate the target state, and maintain the track. Unfortunately, the number of particles is closely related to the computational efficiency of the algorithm. The larger the number of particles, the greater the complexity of the algorithm and the higher the demand for equipment. The measurement-driven approach is an effective way to work out this problem, and is widely used in different filters [28,29].

In order to solve the multi-target tracking problem in case the target detection probability density is unknown, the clutter density is unknown, and the target’s prior position information is lacking, this paper proposes a new robust measurement-driven cardinality balance multi-target multi-Bernoulli (RMD-CBMeMBer) filter. This paper consists of five parts for detailed explanation. In Section 2, the target state is extended, and the extended target state includes target detection probability, kernel state (position, velocity, and angular velocity information), and indicators of clutter and true target. Then, a dynamic model and standard measurement model are presented. In Section 3, the correlation function (CF) is proposed by utilizing the measurement information at the previous moment, and the RMD-CBMeMBer filter is derived in detail based on CF. The particle filter application of RMD-CBMeMBer filter is given in Section 4. In Section 5, a non-linear experiment is set up to verify the tracking performance of the RMD-CBMeMBer filter. Finally, the conclusions are summarized in Section 6.

## 2. System Model

### 2.1. State Motion Model

The traditional single-target state generally only contains such information as the target’s position, velocity, and angular velocity, which is represented by a lowercase letter ${\tilde{x}}_{k}^{i}={\left[x_{k,loc}^{i}y_{k,loc}^{i}v_{x,k}^{i}v_{y,k}^{i}\theta_{k}^{i}\right]}^{T}\in X,i=1,2,\cdot \cdot \cdot ,N_{k}$, where *k* is the time indicator; *i* is the first target at time *k*; X is the space composed of the state cores of all targets. The five elements that make up ${\tilde{x}}_{k}^{i}$ represent the X-axis position, Y-axis position, X-direction speed, Y-direction speed, and turning rate, respectively. In order to solve the multi-target tracking problem with unknown clutter rate and unknown detection probability, the target state is first extended to $x_{k}^{i}=\left[p_{D,k}^{i};{\tilde{x}}_{k}^{i};\Lambda \right],i=1,2,\cdot \cdot \cdot ,N_{k}$. Among them, $p_{D,k}^{i}\in X^{\left(p_{D}\right)}$ represents the detection probability of ${\tilde{x}}_{k}^{i}$, and $X^{\left(p_{D}\right)}$ is the space of target detection probability. Λ∈{0,1} is employed to distinguish between clutter and target, Λ=0 specify that ${\tilde{x}}_{k}^{i}$ is clutter, and Λ=1 indicates that ${\tilde{x}}_{k}^{i}$ is the true target. All single target states at time *k* form a random finite set [1], which is represented by a capital letter $X_{k}={\left\{x_{k}^{i}\right\}}_{i=1}^{N_{k}}\subseteq X^{\left(p_{D}\right)}\times F\left(X\right)\times \left\{0,1\right\}$, where $N_{k}$ is the total number of targets that appear in the observation area at time *k*; F(X) represents a finite subset of elements taken from X; and × represents a Cartesian product. The multi-target kernel state evolves according to the following dynamics model:

(1) $${\tilde{x}}_{k|k+1}^{i}=\left\{\begin{array}{c} F_{k}{\tilde{x}}_{k}^{i}+w_{k};\Lambda =1\\ {\tilde{x}}_{k}^{i}+w_{k};\Lambda =0 \end{array}\right.$$

where $F_{k}$ is the kernel state transition matrix at time *k*, $w_{k}∼N\left(0,\sum \right)$ indicates the white Gaussian noise at time *k*, and ∑ is the corresponding covariance matrix. Note that the prediction of the clutter kernel state is implemented by a general random walk model.

### 2.2. Measurement Model and Bayes Rule

The standard measurement model is based on the following assumptions:

1. Any target $x_{k}^{i}$ that appears in the observation area will produce a unique point measurement value with a detection probability $p_{D,k}^{i}$, or not produce a measurement value with a probability of $\left(1-p_{D,k}^{i}\right)$;
2. The measurement values generated by each true target and the various clutter measurements generated by the sensor are independent of each other;
3. The true but unknown clutter density obeys the Poisson distribution with parameter λ;
4. Given any single target state *x* and a point measurement value *z*, a unique likelihood function can be obtained g(x|z).

With the above assumptions, the measurement values produced by all true targets $X_{k}={\left\{x_{k}^{i}\right\}}_{i=1}^{N_{k}}$ at time *k* can be expressed as a set $Z_{k,T}={\left\{z_{k}^{j}\right\}}_{j=1}^{M_{k,T}}$, where $M_{k,T}\leq N_{k}$ is the number of measurement values produced by the true target. If the measurement values of clutter generated by the sensor at time *k* are composed of $Z_{k,\Gamma }={\left\{z_{k}^{j}\right\}}_{j=1}^{M_{k,\Gamma }}$, then all the measurement values generated at time *k* can be expressed as:

(2) $$Z_{k}=\left(Z_{k,T}\cup Z_{k,\Gamma }\right)={\left\{z_{k}^{j}\right\}}_{j=1}^{M_{k}}\subseteq F\left(Z\right)$$

where $M_{k}$ is the number of all measurements at time *k*, Z is the measurement space composed of all possible measurement values, and F(Z) represents a subset of Z. Note that $Z_{k}$ is also a random finite set.

Suppose f(·|·) and g(·|·) define the multi-target state transition probability density function and multi-target likelihood function, respectively. $\pi_{k-1}\left(\cdot \right)$ represents the posterior probability density function of the multi-target RFS at time k−1. Given the measurement set $Z_{k}$ at time *k*, then the posterior probability density of the multi-target state can be propagated in time according to the Bayes recursion rule [8]:

(3) $$\pi_{k|k-1}\left(X_{k}|Z_{k-1}\right)=\int f\left(X_{k}|X\right)\pi_{k-1}\left(X|Z_{k-1}\right)dX$$

(4) $$\pi_{k}\left(X_{k}|Z_{k}\right)=\frac{g\left(Z_{k}|X_{k}\right)\pi_{k|k-1}\left(X_{k}|Z_{k-1}\right)}{\int g\left(Z_{k}|X\right)\pi_{k|k-1}\left(X|Z_{k-1}\right)dX}$$

## 3. RMD-CBMeMBer Filter

In the standard CBMeMBer filter, the detection probability of the target and the density of the clutter are usually assumed to be known, but in fact they are not. Meanwhile, the newborn BC at all times also obeys a fixed distribution, that is, newborn BC will be distributed in some specific areas. Obviously, this is very unfavorable for tracking targets that may appear at any position at any time. In order to achieve the purpose of tracking the target, it is necessary to completely cover the entire observation area with the newborn BC. This method will cause plenty of system resources to be wasted on target acquisition and track maintenance, and the tracking effect will be severely affected by the distribution of the newborn BC.

In response to the above three problems, a novel RMD-CBMeMBer filter is derived in this section. In the RMD-CBMeMBer filter, the detection probability of the target is modeled as a Beta(α,β)∈[0,1] distribution, and its mean and covariance are $\mu_{B}=\frac{\alpha }{\alpha +\beta }$ and $\nu_{B}=\frac{\alpha \beta }{{\left(\alpha +\beta \right)}^{2}\left(\alpha +\beta +1\right)}$, respectively. Different detection probability models can be obtained by controlling the values of α and β. Meanwhile, the detection probability is extended into the target state, and real-time prediction and update are carried out with the filtering iteration. The clutter is modeled as an independent clutter generator. In the filtering process, each clutter generator is similar to a true target, with its own independent target detection probability, target kernel state transition model, likelihood function, etc. Because there is no correlation between the different clutter generators and between the clutter and the true target, the clutter can be processed separately. The true target and clutter estimation results can be obtained at the same time through filtering. For the new target, we utilize the measurement information at the previous moment to adaptively adjust the distribution of the newborn BC at the subsequent moment, so as to achieve better tracking of the target.

### 3.1. Prediction of RMD-CBMeMBer Filter

In the RMD-CBMeMBer filter, it is assumed that the posterior probability density of multiple targets at time can be expressed as a parameter set:

(5) $$\pi_{k-1|k-1}={\left\{r_{k-1|k-1}^{i},P_{k-1|k-1}^{\Lambda ,i}\right\}}_{i=1}^{M_{k-1|k-1}}$$

where $M_{k-1|k-1}$ is the number of BCs at time k−1, and $r_{k-1|k-1}^{i}$ and $P_{k-1|k-1}^{\Lambda ,i}$ are the existence probability and spatial distribution density of the *i*th BC, respectively. It is worth noting that in the standard CBMeMBer filter, only the state, existence probability and spatial distribution density of the target and clutter need to be predicted. However, in the RMD-CBMeMBer filter, it is necessary to predict the detection probability of the target and clutter as well.

The kernel state prediction of the extended target (the 2–6 dimensions of $x_{k-1}^{i}$) is carried out according to Equation (1). The true target and clutter indicators in the extended target state remain unchanged (the 7 dimension of $x_{k-1}^{i}$). The prediction of the detection probability (the first dimension of $x_{k-1}^{i}$) in the extended target state $x_{k-1}^{i}$ is independent of the kernel state ${\tilde{x}}_{k-1}^{i}$, and is completely controlled by the parameters $\left(\alpha_{k|k-1},\beta_{k|k-1}\right)$ of the Beta distribution:

(6) $$x_{k|k-1}^{i}\left(1\right)=p_{D,k|k-1}^{i}=\mathrm{Beta}\left(\alpha_{k|k-1},\beta_{k|k-1}\right)$$

where $x_{k|k-1}^{i}\left(1\right)$ represents the first dimension of the predicted target state,

(7) $$\alpha_{k|k-1}=\left(\frac{\mu_{k|k-1}\left(1-\mu_{k|k-1}\right)}{\nu_{k|k-1}^{2}}-1\right)\mu_{k|k-1}$$

(8) $$\beta_{k|k-1}=\left(\frac{\mu_{k|k-1}\left(1-\mu_{k|k-1}\right)}{\nu_{k|k-1}^{2}}-1\right)\left(1-\mu_{k|k-1}\right)$$

In the prediction process, the mean value of the Beta probability density function remains unchanged $\mu_{k|k-1}=\mu_{k-1|k-1}$, and the covariance is expanded to $\nu_{k|k-1}=\kappa_{B}\nu_{k-1|k-1}$, $\kappa_{B}>0$ (a typical option is $\kappa_{B}>1$, which means to increase the covariance of the beta distribution).

The predicted multi-target posterior probability density can also be parameterized as:

(9) $$\begin{array}{cc} \pi_{k|k-1} & ={\left\{r_{k|k-1}^{i},P_{k|k-1}^{\Lambda ,i}\right\}}_{i=1}^{M_{k|k-1}}\\ \; & ={\left\{r_{k|k-1}^{i},P_{k|k-1}^{\Lambda ,i}\right\}}_{i=1}^{M_{k-1|k-1}}\cup {\left\{r_{k|k-1,\Gamma }^{i},P_{k|k-1,\Gamma }^{\Lambda ,i}\right\}}_{i=1}^{M_{k|k-1,\Gamma }\left(Z_{k-1}\right)} \end{array}$$

(10) $$r_{k|k-1}^{i}=r_{k-1|k-1}^{i}\sum_{\Lambda \in \{0,1\}}\langle P_{k-1|k-1}^{\Lambda ,i},P_{S}^{\Lambda }\rangle$$

(11) $$\begin{array}{cc} P_{k|k-1}^{\Lambda ,i}\left(x_{k|k-1}\right) & =\frac{\langle f\left(x_{k|k-1}|\cdot \right),P_{k-1|k-1}^{\Lambda ,i}P_{S}^{\Lambda }\rangle }{\langle P_{k-1|k-1}^{\Lambda ,i},P_{S}^{\Lambda }\rangle }\\ \; & =\frac{\langle f\left(p_{D,k|k-1}|\cdot \right)f\left({\tilde{x}}_{k|k-1}|\cdot \right),P_{k-1|k-1}^{\Lambda ,i}P_{S}^{\Lambda }\rangle }{\langle P_{k-1|k-1}^{\Lambda ,i},P_{S}^{\Lambda }\rangle } \end{array}$$

(12) $${\left\{r_{k|k-1,\Gamma }^{i},P_{k|k-1,\Gamma }^{\Lambda ,i}\right\}}_{i=1}^{M_{k|k-1,\Gamma }\left(Z_{k-1}\right)}=\;\mathrm{parameters}\;\mathrm{set}\;\mathrm{of}\;\mathrm{the}\;\mathrm{newborn}\;\mathrm{BC}\;\mathrm{at}\;\mathrm{time}\;k$$

$f(x_{k|k-1}|\cdot )$: the mixed transition density of the target state after expansion;

$f(p_{D,k|k-1}|\cdot )$: the transition density of detection probability, $p_{D,k|k-1}$ is the first dimension of $x_{k|k-1}$;

$f({\tilde{x}}_{k|k-1}|\cdot )$: the transition density of the kernel state;

$P_{S}^{\Lambda }$: survival probability density of the true target (Λ=1) or clutter (Λ=0).

In the RMD-CBMeMBer filter, ${\left\{r_{k|k-1,\Gamma }^{i},P_{k|k-1,\Gamma }^{\Lambda ,i}\right\}}_{i=1}^{M_{k|k-1,\Gamma }\left(Z_{k-1}\right)}$ is no longer given a priori, but is adaptively generated according to the measurement at time k−1. $M_{k|k-1,\Gamma }\left(Z_{k-1}\right)$ is the number of newborn BC. $r_{k|k-1,\Gamma }^{i}=B_{k-1,\Gamma }/M_{k|k-1,\Gamma }$, where $B_{k-1,\Gamma }$ is the expected number of newborn targets, which is usually assumed to be a constant (e.g., $B_{k-1,\Gamma }=0.2$). The state of the different newborn BCs can be generated based on the position of different measurement values. $\mathrm{Supprot}(P_{k|k-1,\Gamma }^{\Lambda ,i})\subseteq \mathrm{supprot}(b\left(\cdot |Z_{k}\right))$, where $b(\cdot |Z_{k})$ is the importance density function of the newborn target. Different from the adaptive birth process in the standard CBMeMBer filter, the newborn target state also contains two extensions—the detection probability and the indicator of the target and clutter in the RMD-CBMeMBer filter. Due to the different characteristics of the target and the clutter, their detection probability may obey the Beta distribution with different parameters. If it is desired to generate the detection probability of BC based on the measurement value, we must first determine whether the measurement value is generated by the true target or a clutter. Given any measurement value $z_{k-1}^{j}\in Z_{k-1}$, we establish the correlation function (CF) between the survival target and the measurement $z_{k-1}^{j}$:

(13) $$CF=\left\{\begin{array}{c} 1;g(x_{k|k-1}^{i}|z_{k-1}^{j})\geq \varepsilon ,\forall i\\ 0;g(x_{k|k-1}^{i}|z_{k-1}^{j})<\varepsilon ,\forall i \end{array}\right.$$

where $g(x_{k|k-1}^{i}|z_{k-1}^{j})$ is the likelihood function, and ε (e.g., ε=0.5) is the threshold. If CF=0, the BC produced by the measurement value $z_{k-1}^{j}$ will be considered as clutter, and Λ=0 is assigned. However, it is worth noting that the newborn target at the last moment will not have a predicted state at the current moment (Because there is no measurement information of the newborn target at the moment before the newborn target appears, that is, there is no corresponding BC at the moment when the newborn target appears, so there is no predicted state of the newborn target at the current moment). When CF=0 is established, it may also be a potential target. Therefore, if CF=0 is obtained from the measurement $z_{k-1}^{j}$, it is also necessary to generate a BC with Λ=1 according to $z_{k-1}^{j}$ to track the newborn target. Meanwhile, the detection probability of the BC can be generated by employing different detection probability models according to the value of Λ. If CF=1, then the BC generated by utilizing the measurement $z_{k-1}^{j}$ will be considered as a true target, and Λ=1 is assigned. However, at this time, the BC generated by the measurement $z_{k-1}^{j}$ needs to be fused with the ith surviving BC. In particle applications, fusion is represented as the union of particles and weights, and the survival probability of BC is accumulated.

### 3.2. Update of RMD-CBMeMBer Filter

Given the measurement set $Z_{k}$ at time, the posterior probability density of the RMD-CBMeMBer filter can be expressed as the detected and undetected parts:

(14) $$\pi_{k|k}={\left\{r_{U,k|k}\left(z\right),P_{U,k|k}^{\Lambda }\left(\cdot ;z\right)\right\}}_{z_{k}\in Z_{k}}\cup {\left\{r_{L,k}^{i},P_{L,k}^{\Lambda ,i}\right\}}_{i=1}^{M_{k|k-1}}$$

where $r_{U,k|k}\left(z\right)$ and $P_{U,k|k}^{\Lambda }\left(\cdot ;z\right)$ indicate the detected target (the existence probability and spatial distribution probability density that need to be updated with the measured *z*), and $r_{L,k}^{i}$ and $P_{L,k}^{\Lambda ,i}$ indicate the undetected target (the existence probability and the spatial distribution probability density also need to be updated, but there is no need to use the measurement because of missed detection).

(15) $$r_{U,k|k}=\sum_{\Lambda \in \{0,1\}}r_{U,k|k}^{\Lambda }$$

(16) $$r_{U,k|k}^{\Lambda }\left(z\right)=\frac{\sum_{i=1}^{M_{k|k-1}}\frac{r_{k|k-1}^{i}\left(1-r_{k|k-1}^{i}\right)\langle P_{k|k-1}^{\Lambda ,i},g_{k}^{\Lambda }\left(z|\cdot \right)p_{D,k|k-1}^{\Lambda ,i}\rangle }{{\left(1-r_{k|k-1}^{i}\sum_{\Lambda^{\prime }\in \left\{0,1\right\}}\langle P_{k|k-1}^{\Lambda^{\prime },i},p_{D,k|k-1}^{\Lambda^{\prime },i}\rangle \right)}^{2}}}{\sum_{i=1}^{M_{k|k-1}}\frac{r_{k|k-1}^{i}\sum_{\Lambda^{\prime }\in \left\{0,1\right\}}\langle P_{k|k-1}^{\Lambda^{\prime },i},g_{k}^{\Lambda^{\prime }}\left(z|\cdot \right)p_{D,k|k-1}^{\Lambda^{\prime },i}\rangle }{1-r_{k|k-1}^{i}\sum_{\Lambda^{\prime }\in \left\{0,1\right\}}\langle P_{k|k-1}^{\Lambda^{\prime },i},p_{D,k|k-1}^{\Lambda^{\prime },i}\rangle }}$$

(17) $$P_{U,k|k}^{\Lambda }\left(\tilde{x};z\right)=\frac{\sum_{i=1}^{M_{k|k-1}}\frac{r_{k|k-1}^{i}}{1-r_{k|k-1}^{i}}P_{k|k-1}^{\Lambda ,i}g_{k}^{\Lambda }\left(z|\tilde{x}\right)p_{D,k|k-1}^{\Lambda ,i}}{\sum_{\Lambda^{\prime }\in \left\{0,1\right\}}\sum_{i=1}^{M_{k|k-1}}\frac{r_{k|k-1}^{i}}{1-r_{k|k-1}^{i}}\langle P_{k|k-1}^{\Lambda^{\prime },i},g_{k}^{\Lambda^{\prime }}\left(z|\cdot \right)p_{D,k|k-1}^{\Lambda^{\prime },i}\rangle }$$

(18) $$r_{L,k|k}^{i}=\sum_{\Lambda \in \{0,1\}}r_{L,k|k}^{\Lambda ,i}$$

(19) $$r_{L,k|k}^{\Lambda ,i}=\frac{r_{k|k-1}^{i}\langle P_{k|k-1}^{\Lambda ,i},1-p_{D,k|k-1}^{\Lambda ,i}\rangle }{1-r_{k|k-1}^{i}\sum_{\Lambda^{\prime }\in \left\{0,1\right\}}\langle P_{k|k-1}^{\Lambda^{\prime },i},p_{D,k|k-1}^{\Lambda^{\prime },i}\rangle }$$

(20) $$P_{L,k|k}^{\Lambda ,i}=\frac{\left(1-p_{D,k|k-1}^{\Lambda ,i}\right)P_{k|k-1}^{\Lambda ,i}}{\sum_{\Lambda^{\prime }\in \left\{0,1\right\}}\langle P_{k|k-1}^{\Lambda^{\prime },i},1-p_{D,k|k-1}^{\Lambda^{\prime },i}\rangle }$$

Obviously, the update method of the target’s existence probability and spatial distribution probability density is closely related to whether it is detected. If the target is not detected, the target’s existence probability and spatial distribution probability density need to be updated according to Equations (18)–(20). At this time, only the missed detection probability $\left(1-p_{D}\right)$ is taken into account. On the contrary, the target’s existence probability and spatial distribution probability density need to be updated according to Equations (15)–(17). At this time, the likelihood function and the detection probability of the target should be considered at the same time.

### 3.3. State Extraction

Since the extended target state can distinguish the true target and the clutter, the state can be extracted from the estimation results of the true target and the clutter generator, respectively. Assume that the updated multi-target posterior probability density can be rewritten as:

(21) $$\pi_{k|k}={\left\{r_{U,k|k}\left(z\right),P_{U,k|k}^{\Lambda }\left(\cdot ;z\right)\right\}}_{z_{k}\in Z_{k}}\cup {\left\{r_{L,k}^{i},P_{L,k}^{\Lambda ,i}\right\}}_{i=1}^{M_{k|k-1}}={\left\{r_{k}^{i},P_{k}^{\Lambda ,i}\right\}}_{i=1}^{M_{k|k}}$$

where,

(22) $$r_{k}^{i}=r_{k}^{\Lambda =0,i}+r_{k}^{\Lambda =1,i}$$

Then, the estimated result of the number of true targets is:

(23) $$N_{k}^{\Lambda =1}=\sum_{i=1}^{M_{k|k}}r_{k}^{\Lambda =1,i}$$

The state and the detection probability of the true target can be extracted from the distribution $P_{k}^{\Lambda =1,i}\left(x\right)$ and the extended target state at time *k*, respectively. The average clutter rate is estimated as:

(24) $$\lambda_{k}=\sum_{i=1}^{M_{k|k}}r_{k}^{\Lambda =0,i}\int p_{D,k|k}^{\Lambda =0,i}P_{k}^{\Lambda =0,i}\left(x\right)$$

## 4. Particle Implementation

Particle filter is an important way of implementing filters, which uses a cluster of weighted particles to describe the target state. The goal of estimating the target state is achieved by updating the weight and state of particles. This section derives the particle application of the RMD-CBMeMBer filter in detail based on the previous section. Assume that the multi-target posterior density at time k−1 can be expressed as:

(25) $$\pi_{k-1|k-1}={\left\{r_{k-1|k-1}^{i},P_{k-1|k-1}^{\Lambda ,i}\right\}}_{i=1}^{M_{k-1|k-1}}$$

where,

(26) $$r_{k-1|k-1}^{i}=\sum_{\Lambda \in \{0,1\}}r_{k-1|k-1}^{\Lambda ,i}$$

(27) $$P_{k-1|k-1}^{\Lambda ,i}=\sum_{n=1}^{J_{k-1|k-1}^{\Lambda ,i}}w_{n,k-1}^{\Lambda ,i}\delta_{x_{n,k-1|k-1}^{i}}\left(x\right)$$

where $x_{n,k-1|k-1}^{i}$ represents the *n*th particle describing the *i*th BC, and $w_{n,k-1}^{\Lambda ,i}$ is the corresponding weight; $\delta_{X}\left(Y\right)\overset{\Delta }{\;=}\left\{\begin{array}{c} 1,\;\;\;\;\mathrm{if}\;X=Y\;\;\;\;\;\;\;\\ 0,\mathrm{otherwise} \end{array}\right.$ defines the generalized Kronecker function; $J_{k-1|k-1}^{\Lambda ,i}$ represents the number of all particles carrying indicator Λ in the *i*th Bernoulli component; $\delta_{x_{n,k-1|k-1}^{i}}\left(x\right)=\delta_{x_{n,k-1|k-1}^{i}\left(1\right)}\left(x\left(1\right)\right)\times \delta_{{\tilde{x}}_{n,k-1|k-1}^{i}}\left(\tilde{x}\right)\times \delta_{x_{n,k-1|k-1}^{i}\left(7\right)}\left(x\left(7\right)\right)$, the three items on the right correspond to the detection probability of the target, the kernel state, and the indicator of the true target and clutter. Among them, $x_{n,k-1|k-1}^{i}\left(j\right)$ indicates the *j*th dimension of the particle $x_{n,k-1|k-1}^{i}$.

### 4.1. Prediction

In the prediction process, the kernel state of the particles is predicted by using Equation (1), the detection probability of the particles is achieved by employing Equations (6)–(8), and the indicators of the target and clutter in the particle states remain unchanged before and after the prediction. Finally, the predicted states of the extended particle $x_{n,k-1|k-1}^{i}\to x_{n,k|k-1}^{i}$ can be obtained. The particle weight is predicted as:

(28) $$w_{n,k|k-1}^{\Lambda ,i}=\frac{w_{n,k-1|k-1}^{\Lambda ,i}f\left(x_{n,k|k-1}^{i}|\cdot \right)P_{S}^{\Lambda }}{\sum_{n=1}^{J_{k|k-1}^{\Lambda ,i}}w_{n,k-1|k-1}^{\Lambda ,i}P_{S}^{\Lambda }}=\frac{w_{n,k-1|k-1}^{\Lambda ,i}\left(f\left(p_{D,n,k|k-1}^{\Lambda ,i}|\cdot \right)f\left({\tilde{x}}_{n,k|k-1}^{i}|\cdot \right)\right)P_{S}^{\Lambda }}{\sum_{n=1}^{J_{k|k-1}^{\Lambda ,i}}w_{n,k-1|k-1}^{\Lambda ,i}P_{S}^{\Lambda }}$$

where the denominator is a normalized constant; $f(p_{D,n,k|k-1}^{\Lambda ,i}|\cdot )$ is the detection probability transition density; $f({\tilde{x}}_{n,k|k-1}^{i}|\cdot )$ represents the transition density of the kernel state; $J_{k|k-1}^{\Lambda ,i}=J_{k-1|k-1}^{\Lambda ,i}$ is the number of particles contained in the *i*th BC after prediction. Then, the predicted spatial distribution density and existence probability of the *i*th surviving BC are:

(29) $$P_{k|k-1}^{\Lambda ,i}=\sum_{n=1}^{J_{k|k-1}^{\Lambda ,i}}w_{n,k|k-1}^{\Lambda ,i}\delta_{x_{n,k|k-1}^{i}}\left(x\right)$$

(30) $$r_{k|k-1}^{i}=r_{k-1|k-1}^{i}\sum_{\Lambda \in \{0,1\}}\sum_{n=1}^{J_{k|k-1}^{\Lambda ,i}}w_{n,k|k-1}^{\Lambda ,i}$$

The predicted multi-object posterior density is expressed as:

(31) $$\begin{array}{cc} \pi_{k|k-1} & ={\left\{r_{k|k-1}^{i},P_{k|k-1}^{\Lambda ,i}\right\}}_{i=1}^{M_{k|k-1}}\\ \; & ={\left\{r_{k|k-1}^{i},P_{k|k-1}^{\Lambda ,i}\right\}}_{i=1}^{M_{k-1|k-1}}\cup {\left\{r_{k|k-1,\Gamma }^{i},P_{k|k-1,\Gamma }^{\Lambda ,i}\right\}}_{i=1}^{M_{k|k-1,\Gamma }\left(Z_{k-1}\right)} \end{array}$$

where $r_{k|k-1,\Gamma }^{i},P_{k|k-1,\Gamma }^{\Lambda ,i}$ and $M_{k|k-1,\Gamma }$ represent the existence probability and the spatial distribution density of the *i*th BC and the number of newborn BCs at time *k*, respectively. In particle implementation, the measurement information at the previous moment is also utilized to create new particles. The value of $r_{k|k-1,\Gamma }^{i}=B_{k-1,\Gamma }/M_{k|k-1,\Gamma }\left(Z_{k-1}\right)$ and $M_{k|k-1,\Gamma }\left(Z_{k-1}\right)$ is related to the number of elements in $Z_{k-1}$. $P_{k|k-1,\Gamma }^{\Lambda ,i}$ is described by a cluster of particles $\;\;\;{\left\{\;\;\;x_{\Gamma ,n,k|k-1}^{i},w_{\Gamma ,n,k|k-1}^{\Lambda ,i}\right\}}_{n=1}^{J_{\Gamma ,k|k-1}^{\Lambda ,i}}$:

(32) $$P_{k|k-1,\Gamma }^{\Lambda ,i}=\sum_{n=1}^{J_{\Gamma ,k|k-1}^{\Lambda ,i}}w_{\Gamma ,n,k|k-1}^{\Lambda ,i}\delta_{x_{\Gamma ,n,k|k-1}^{i}}\left(x\right)$$

The new particle generation method follows the measurement-driven principle in Section 3.1. Note that the correlation function (CF) between the survival target and measurement $z_{k-1}^{j}$ changes to:

(33) $$CF=\left\{\begin{array}{c} 1;\sum_{n=1}^{J_{k|k-1}^{\Lambda ,i}}g(x_{n,k|k-1}^{i}|z_{k-1}^{j})\geq \varepsilon ,\forall i\\ 0;\sum_{n=1}^{J_{k|k-1}^{\Lambda ,i}}g(x_{n,k|k-1}^{i}|z_{k-1}^{j})<\varepsilon ,\forall i \end{array}\right.$$

The kernel state of the newborn particles is assumed to be Gaussian distribution N(m,P), where the position information in the mean *m* is initialized according to the position of the measurement, the velocity and angular velocity information are initialized to 0, and the covariance *P* is a given value.

### 4.2. Update

Given the measurement set $Z_{k}={\left\{z_{k}^{i}\right\}}_{i=1}^{M_{k}}$ generated by the sensor at time *k*, the posterior probability density of the RMD-CBMeMBer filter can be expressed as a parameter set:

(34) $$\pi_{k|k}={\left\{r_{k|k}^{i},P_{k|k}^{\Lambda ,i}\right\}}_{i=1}^{M_{k|k}}={\left\{r_{U,k|k}\left(z\right),P_{U,k|k}^{\Lambda }\left(\cdot ;z\right)\right\}}_{z_{k}\in Z_{k}}\cup {\left\{r_{L,k}^{i},P_{L,k}^{\Lambda ,i}\right\}}_{i=1}^{M_{k|k-1}}$$

where,

(35) $$r_{U,k|k}\left(z\right)=\sum_{\Lambda \in \{0,1\}}r_{U,k|k}^{\Lambda }\left(z\right)$$

(36) $$r_{U,k|k}^{\Lambda }\left(z\right)=\frac{\sum_{i=1}^{M_{k|k-1}}\frac{r_{k|k-1}^{i}\left(1-r_{k|k-1}^{i}\right)\rho_{U,k|k}^{\Lambda ,i}\left(z\right)}{{\left(1-r_{k|k-1}^{i}\sum_{\Lambda^{\prime }\in \left\{0,1\right\}}\rho_{L,k|k}^{\Lambda^{\prime },i}\left(z\right)\right)}^{2}}}{\sum_{i=1}^{M_{k|k-1}}\frac{r_{k|k-1}^{i}\sum_{\Lambda^{\prime }\in \left\{0,1\right\}}\rho_{U,k|k}^{\Lambda^{\prime },i}\left(z\right)}{1-r_{k|k-1}^{i}\sum_{\Lambda^{\prime }\in \left\{0,1\right\}}\rho_{L,k|k}^{\Lambda^{\prime },i}\left(z\right)}}$$

(37) $$P_{U,k|k}^{\Lambda }\left(\tilde{x};z\right)=\sum_{i=1}^{M_{k|k-1}}\sum_{n=1}^{J_{k|k-1}^{\Lambda ,i}}w_{U,n,k|k}^{\Lambda ,i}\left(z\right)\delta_{x_{n,k|k-1}^{i}}\left(x\right)$$

(38) $$r_{L,k|k}^{i}=\sum_{\Lambda \in \{0,1\}}r_{L,k|k}^{\Lambda ,i}$$

(39) $$r_{L,k|k}^{\Lambda ,i}=r_{k|k-1}^{i}\frac{{\tilde{\rho }}_{L,k|k}^{\Lambda ,i}-\rho_{L,k|k}^{\Lambda ,i}}{1-r_{k|k-1}^{i}\sum_{\Lambda^{\prime }\in \left\{0,1\right\}}\rho_{L,k|k}^{\Lambda^{\prime },i}}$$

(40) $$P_{L,k|k}^{\Lambda ,i}\left(x\right)=\sum_{n=1}^{J_{k|k-1}^{\Lambda ,i}}w_{L,n,k|k}^{\Lambda ,i}\left(z\right)\delta_{x_{n,k|k-1}^{i}}\left(x\right)$$

where,

(41) $${\tilde{\rho }}_{L,k|k}^{\Lambda ,i}=\sum_{n=1}^{J_{k|k-1}^{\Lambda ,i}}w_{n,k|k-1}^{\Lambda ,i}$$

(42) $$\rho_{L,k|k}^{\Lambda ,i}=\sum_{n=1}^{J_{k|k-1}^{\Lambda ,i}}w_{n,k|k-1}^{\Lambda ,i}p_{D,n,k|k-1}^{\Lambda ,i}$$

(43) $$w_{L,n,k|k}^{\Lambda ,i}=w_{n,k|k-1}^{\Lambda ,i}\left(1-p_{D,n,k|k-1}^{\Lambda ,i}\right)/\sum_{\Lambda \in \{0,1\}}\sum_{n=1}^{J_{k|k-1}^{\Lambda ,i}}w_{n,k|k-1}^{\Lambda ,i}\left(1-p_{D,n,k|k-1}^{\Lambda ,i}\right)\;$$

(44) $$\rho_{U,k|k}^{\Lambda ,i}=\sum_{n=1}^{J_{k|k-1}^{\Lambda ,i}}w_{n,k|k}^{\Lambda ,i}g_{k}^{\Lambda }\left(z|x_{n,k|k-1}^{i}\right)p_{D,n,k|k-1}^{\Lambda ,i}$$

(45) $${\tilde{w}}_{U,n,k|k}^{\Lambda ,i}\left(\left[z\right]\right)=w_{U,n,k|k}^{\Lambda ,i}\left(z\right)/\sum_{\Lambda \in \{0,1\}}\sum_{j=1}^{M_{k|k-1}}\sum_{n=1}^{J_{k|k-1}^{\Lambda ,j}}w_{U,n,k|k}^{\Lambda ,j}\left(z\right)\;$$

(46) $$w_{U,n,k|k}^{\Lambda ,i}\left(z\right)=\frac{r_{k|k-1}^{i}}{1-r_{k|k-1}^{i}}w_{n,k|k-1}^{\Lambda ,i}g_{k}^{\Lambda }\left(z|x_{n,k|k-1}^{i}\right)p_{D,n,k|k-1}^{\Lambda ,i}$$

The state of the *i*th true target at time *k* after the update is:

(47) $$x_{k}^{\Lambda =1,i}=\sum_{\Lambda =1}\sum_{n=1}^{J_{k|k-1}^{\Lambda ,i}}w_{n,k|k}^{\Lambda ,i}x_{n,k|k-1}^{i}$$

The first dimension $x_{k}^{\Lambda =1,i}\left(1\right)$ of the extended target state $x_{k}^{\Lambda =1,i}$ is the estimated detection probability. The estimated number of the true targets at time *k* is Equation (23). The estimated average clutter rate at time *k* is:

(48) $$\lambda_{k}=\sum_{i=1}^{M_{k|k}}r_{k}^{\Lambda =0,i}\sum_{n=1}^{J_{k|k}^{i}}w_{n,k|k}^{\Lambda ,i}p_{D,n,k|k}^{\Lambda =0,i}$$

In the particle implementation of the RMD-CBMeMBer filter, the particle degradation problem also exists [8]. Therefore, the re-sampling [8] step is performed for each BC in turn after the update. The number of particles of each BC after resampling is $\min\{\max\left\{r_{k,\Gamma }^{\Lambda ,i}J_{k|k}^{\left(i\right)},J_{\min}\right\},J_{\max}\}$ ($J_{\min}$ is the minimum number of particles, e.g., 3500, and $J_{\max}$ is the maximum number of particles, e.g., 5000). In addition, the number of Bernoulli components will increase exponentially with the iteration of the filter, so it is necessary to set the threshold (e.g., $10^{-3}$) of the existence probability of BCs as in [8] to perform pruning operations to avoid unlimited increase in the number of BCs.

## 5. Numerical Experiment

### 5.1. Experimental Environment

**Figure 1:**
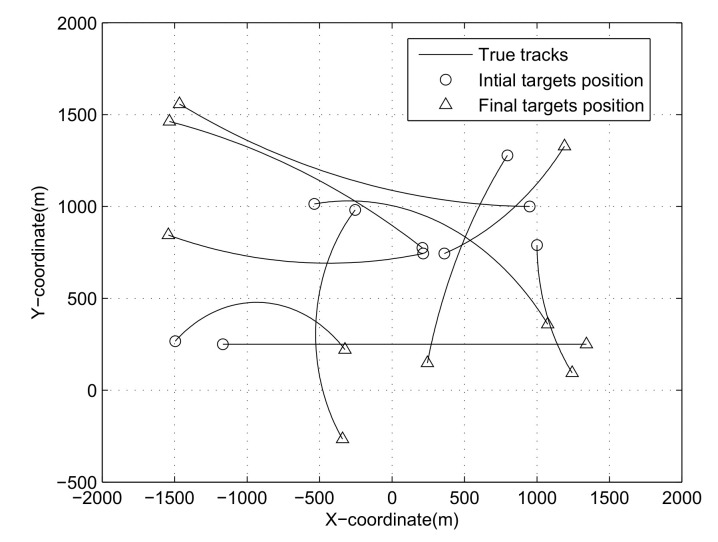
True trajectories.

There are 10 targets in the numerical experiment. The observation area is [−2000m,2000m]×[−500m,2000m] (the observation area in the X direction and in the Y direction). The sampling interval is T=1s. The survival probability of the target is set to be $P_{S}=0.99$. The true but unknown detection probability is set to be $p_{D}=0.8$. Figure 1 shows the true trajectories of all targets, and Table 1 gives the initial state information of the 12 targets and the existence time period information of each target. The “Initial state” in Table 1 contains 5 dimensions, which are the initial position of the target in the X direction, the initial position in the Y direction, the initial velocity in the X direction, the initial velocity in the Y direction, and the initial angular velocity. The target existence time period (“Time interval” in Table 1) is divided into two dimensions. The first dimension represents the moment when the target appears (birth time), and the second dimension is the moment when the target disappears (death time).

The 1st dimension of the target state $x_{k}^{i}$ is that the detection probability obeys the Beta distribution; the 2–6th dimensions of $x_{k}^{i}$ are the position and velocity information. The kernel state transition of the true target in the experiment obeys:

(49) $$x_{k}\left(2:6\right)=\left|\begin{array}{ccccc} 1 & 0 & \frac{\sin\theta T}{\theta } & -\frac{1-\cos\theta T}{\theta } & 0\\ 0 & 1 & \frac{1-\cos\theta T}{\theta } & \frac{\sin\theta T}{\theta } & 0\\ 0 & 0 & \cos\theta T & \frac{\sin\theta T}{\theta } & 0\\ 0 & 0 & \sin\theta T & -\sin\theta T & 0\\ 0 & 0 & 0 & 0 & 1 \end{array}\right|x_{k-1}\left(2:6\right)+v_{k}$$

where, $x_{k}\left(2:6\right)$ represents the 2–6th dimensions of $x_{k}^{i}$, $\sigma \;=\;\sqrt{15}$ and T=1s. $\theta =x_{k-1}\left(6\right)$ is the angular velocity. The white Gaussian process noise $v_{k}$ is independent of the model, and the covariance matrix is *Q*.

(50) $$Q=\sigma^{2}\times \left|\begin{array}{ccccc} \frac{T^{3}}{3} & 0 & \frac{T^{2}}{2} & 0 & 0\\ 0 & \frac{T^{3}}{3} & 0 & \frac{T^{2}}{2} & 0\\ \frac{T^{2}}{2} & 0 & T & 0 & 0\\ 0 & \frac{T^{2}}{2} & 0 & T & 0\\ 0 & 0 & 0 & 0 & \frac{\pi }{60} \end{array}\right|$$

The evolution of the clutter kernel state in the experiment obeys the random walk model:

(51) $$x_{k}\left(2:6\right)=x_{k-1}\left(2:6\right)+v_{k}$$

For convenience, the covariance matrix for the process noise of the true target kernel state and the clutter kernel state is set to be the same. The sensor measurement model includes angle and distance:

(52) $$z_{k}^{i}=\left|\begin{array}{c} \arctan\frac{{\tilde{x}}_{k}^{i}\left(4\right)-d_{y}^{s}}{{\tilde{x}}_{k}^{i}\left(2\right)-d_{x}^{s}}\\ \sqrt{{\left({\tilde{x}}_{k}^{i}\left(2\right)-d_{x}^{s}\right)}^{2}+{\left({\tilde{x}}_{k}^{i}\left(4\right)-d_{y}^{s}\right)}^{2}} \end{array}\right|+w_{k}^{i}$$

where, $\left[d_{x}^{s},d_{y}^{s}\right]=\left[0\;m,0\;m\right]$ is the position of the sensor, and only one is set in the experiment. $w_{k}^{i}$ is white Gaussian noise with a standard deviation of $\mathrm{diag}(\left[\frac{\pi }{180}\;\mathrm{rad},5\;m\right])$. For true targets, the probability of existence of the newborn BC at each moment is $r^{\left(i\right)}=0.2,i=1,2,\cdot \cdot \cdot$. The first dimension of the newborn target states in the BCs is the detection probability of the target that obeys the Beta distribution β(·;2,10). The 7th dimension of the true target state (indicator for the true target and the clutter) is 1. The 2–6th dimensions of the target state are the target position, velocity, and angular velocity information. The mean of the position in the newborn target state is generated according to the measurement, and the mean of the velocity and the mean of the angular velocity are initialized to 0. The covariance matrix of the newborn target state is set as:

(53) $$P^{\left(i\right)}\;=\;\mathrm{diag}(\;\;\;\left[\;\;\;25{\;m}^{2}\;25{\;m}^{2}\cdot s^{-2}\;25{\;m}^{2}\;25{\;m}^{2}\cdot s^{-2}\;\frac{\pi }{60}\;\mathrm{ra}d^{2}\;\;\;\right]\;\;\;),\;i\;=\;1,\;2,\cdot \cdot \cdot ,\;4$$

Similarly, the mean of the position of the clutter is also generated based on the measurement, and the mean of velocity and angular velocity are both initialized to 0. The first dimension (detection probability) of the clutter state obeys the beta distribution with parameter β(·;5,7). The 7th dimension (indicator of ture target and clutter) are 0. For convenience, the covariance matrix of the clutter kernel state (2–6th dimensions) is also set to be Equation (53). The performance of the proposed algorithm is evaluated by utilizing the optimal subpattern assignment [30] (OSPA) distance.

### 5.2. Experimental Analysis

**Figure 2:**
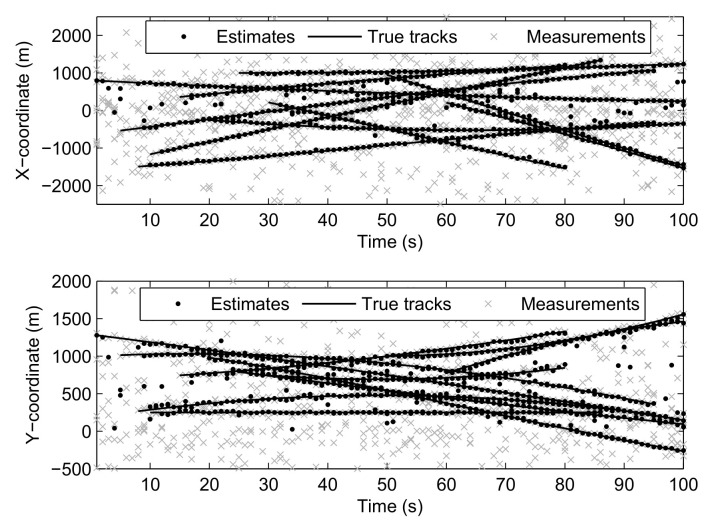
Location estimation of multi-target.

Figure 2 shows the effect of RMD-CBMeMBer filter on multi-target position estimation when the detection probability and clutter rate are both unknown. It can be seen that the RMD-CBMeMBer filter proposed in this paper can achieve accurate estimation of the position for multi-target tracking with unknown background.

**Figure 3:**
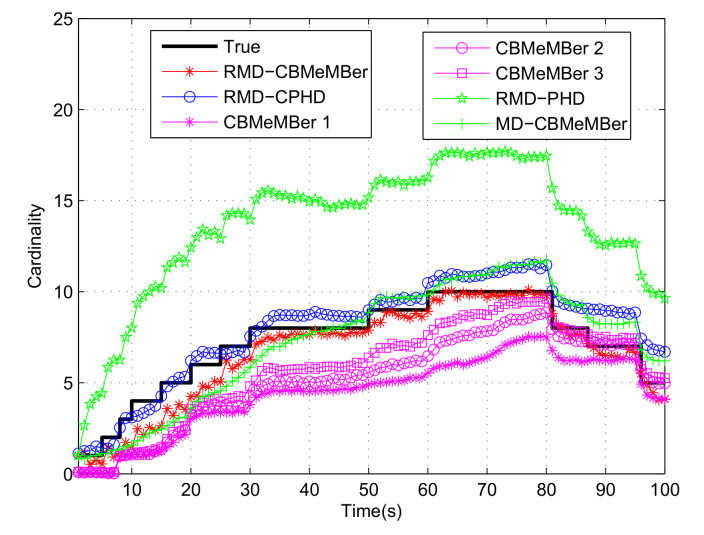
Cardinality estimation of multi-target.

Figure 3 shows the estimation results of different filters on the number of targets when the detection probability and the clutter rate are both unknown. RMD-CPHD refers to the robust measurement-driven CPHD filter, which also uses the measurement-driven newborn strategy mentioned in this paper. RMD-PHD refers to the robust measurement-driven PHD filter. CBMeMBer1, CBMeMBer2 and CBMeMBer3 represent the standard CBMeMBer filter. The number of newborn BCs in these three filters is 200, 500, and 1000, respectively. MD-CBMeMBer represents the measurement-driven CBMeMBer filter. In the MD-CBMeMBer filter, the detection probability and the clutter rate are known a priori. It can be seen from Figure 3:

1. The RMD-CBMeMBer filter proposed in this paper (each newborn BC contains 200 particles) has a certain delay in the estimation of the number of targets. The reason for this phenomenon is that the newborn BCs is generated based on the measurement information at the previous moment. When the target appears at time *k*, the measurement information of the newborn target at time *k* cannot be obtained at time k−1. The newborn BC at time *k* does not contain the information of the newborn target at time *k*, which makes the newborn target unable to be captured and tracked in real time at time *k*. However, the newborn BC at time k+1 contains the information of the newborn target at time *k*, so the newborn target at time *k* can be successfully captured and tracked at time k+1;
2. Compared with the RMD-CPHD filter, in the stage where the target is frequently newborn (such as 1–30 s), the RMD-CBMeMBer filter is slightly worse in cardinality estimation, which is caused by the essential difference between the two filters in the realization process. The RMD-CBMeMBer filter generates a BC based on each measurement to characterize the target, and the number of particles in each BC is 200. The RMD-CPHD filter also generates 200 particles based on each measurement, but it estimates the target state after all particles are predicted, updated, and clustered. Its estimated performance is also related to the total number of particles at each moment. For example, a total of 10 measurements are used to generate particles at time *k*, and then a total of 2000 particles will participate in the prediction update. If there are 3 true targets and 2 false targets at time *k*, the number of particles used to describe each target in the RMD-CBMeMBer filter is 200. However, the number of particles used to describe each target after clustering in the RMD-CPHD filter can be far greater than 200. However, the total number of targets appearing in the observation area stabilizes (e.g., 60–80 s). After filtering iterations, most of the particles in the BC employed to describe the target state have larger weight, and the target state estimation result is less affected by the number of particles. At this time, the performance of the RMD-CBMeMBer filter in cardinality estimation is significantly better than that of the RMD-CPHD filter;
3. The RMD-CBMeMBer filter is superior to the CBMeMBer filter in terms of cardinality (number of targets) estimation. The reason is that the CBMeMBer filter uses a fixed position and a fixed distribution to generate BC, which makes the target difficult to capture and track and the target can be lost easily. Although the CBMeMBer filter can slowly approximate the true value in cardinality estimation as the number of particles increases, its computational complexity will increase considerably;
4. The RMD-CBMeMBer filter is significantly better than the RMD-PHD filter in cardinality estimation. Because the RMD-PHD filter only transmits the first moment of the posterior probability density of multiple targets, without considering the cardinality distribution of the target. Therefore, it is easy for RMD-PHD filter to overestimate the number of targets;
5. It can be seen from Figure 3 that the MD-CBMeMBer filter cannot accurately predict the cardinality of the target in the stage where new targets frequently appear (0–30 s). The reason for the loss of the target is the same as that of the RMD-CBMeMBer filter. However, the performance of the MD-CBMeMBer filter is better than the RMD-CBMeMBer filter in the stage when the target number is relatively stable (30–80 s). The reason for this phenomenon is that the detection probability of the target and the background false alarm rate in the MD-CBMeMBer filter are known.

**Figure 4:**
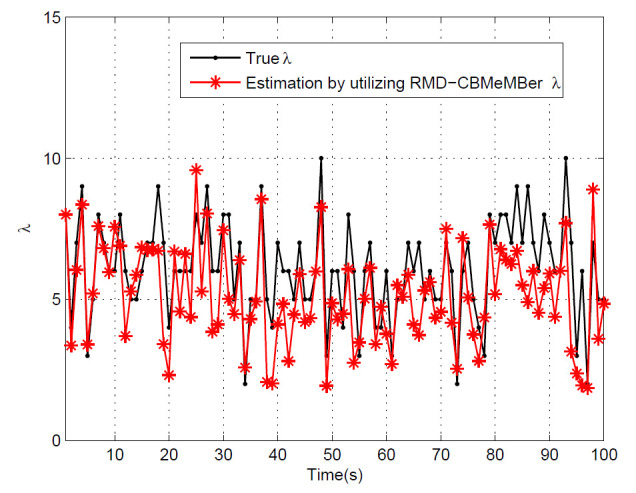
Average clutter rate estimation for RMD-CBMeMBer filter.

Figure 4 shows the estimation results of the average clutter rate of the RMD-CBMeMBer filter. It can be observed that the RMD-CBMeMBer filter can effectively estimate the average clutter rate at different times. Even if the average clutter rate at different times cannot be accurately estimated, the trend of the true clutter rate can be obtained.

**Figure 5:**
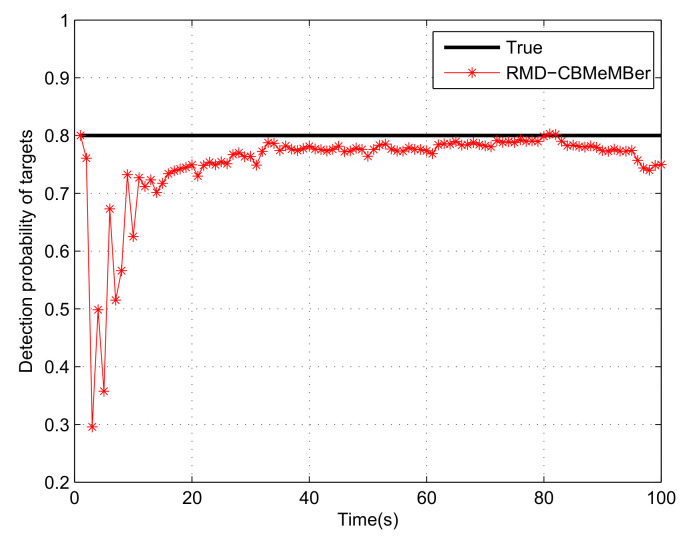
Detection probability estimation for the RMD-CBMeMBer filter.

Figure 5 is the estimation result of the RMD-CBMeMBer filter for the detection probability of the true target. It can be seen that the RMD-CBMeMBer filter cannot accurately estimate the detection probability of the target at the initial few moments. However, the true value of the target detection probability will be achieved with the iteration of time by utilizing the RMD-CBMeMBer filter. In addition, the detection probability estimation result of the target will have an inflection point in the vicinity of the target’s death time (such as 80 s, 95 s, and 96 s). This is because if the BC used to describe the target disappears after the target disappears, the existence probability of the BC needs to be smaller than the threshold, which requires 1–2 s. In this process, the estimated value of the detection probability of the target has an inflection point due to the disappearance of the target and the lack of measurement information.

**Figure 6:**
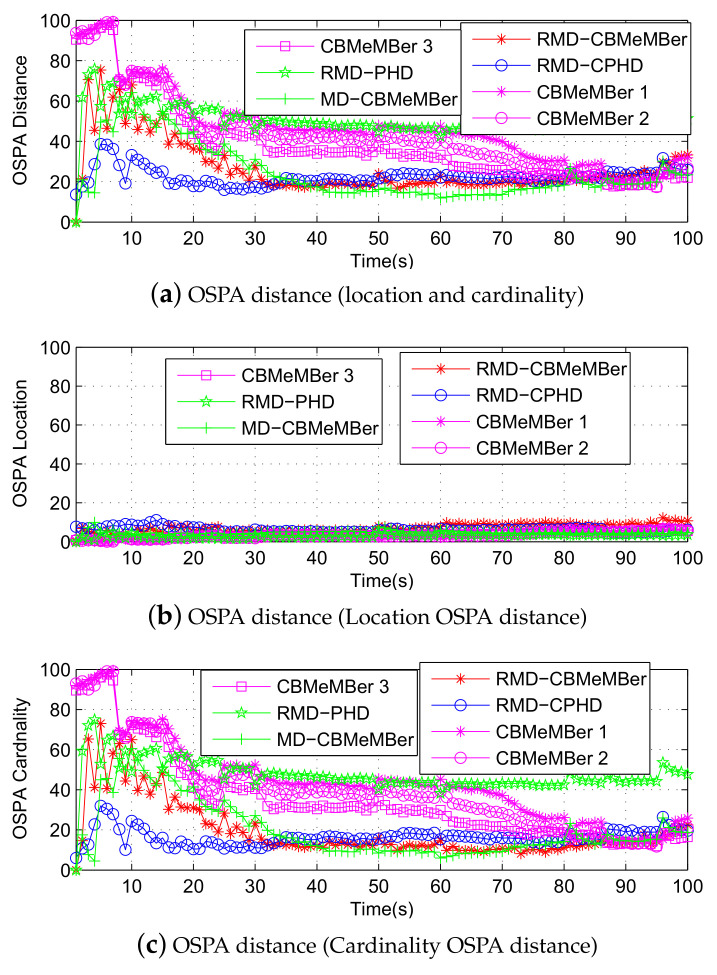
OSPA error estimation(100 MC).

Figure 6a–c are the OSPA distance, the target location OSPA distance and the target cardinality OSPA distance, respectively. Among them, “OSPA Distance” means that the target position estimation and target number estimation error are considered comprehensively. “OSPA Location” represents the target location estimation error, and “OSPA Cardinality” indicates that the estimation error for the number of the target. It can be seen from Figure 6b that the performance of the RMD-CBMeMBer filter is slightly worse than that of other filters in target location estimation. The RMD-CBMeMBer filter is worse than the RMD-CPHD filter in terms of target location estimation. This is because the RMD-CBMeMBer filter adds new particles (measurement-driven) to the surviving BC to describe the target at each moment, and the newly added particles are random to some degree. The RMD-CPHD filter obtains the estimated target state after prediction, update, and clustering, so its estimation performance is less affected by the newborn particles. The reason why the RMD-CBMeMBer filter in target location estimation is inferior to the CBMeMBer filter is similar.

It can be seen from Figure 6c that the RMD-CBMeMBer filter is inferior to the RMD-CPHD filter in terms of target number estimation in the early stage of tracking. However, as time goes by, the performance of the former will gradually surpass the latter in terms of target number estimation. Compared with the RMD-CBMeMBer filter and AR-CPHD filter, the performance of the CBMeMBer filter in the estimation of target number will be severely restricted by the number of particles, and it is difficult to obtain an ideal result. Figure 6a comprehensively considers the estimation error of the target’s position and number. As can be seen, the performance of the RMD-CBMeMBer filter in multi-target state estimation is significantly better than that of the CBMeMBer filter. Compared with the RMD-CPHD filter, the RMD-CBMeMBer filter will gradually be superior to the RMD-CPHD filter with the iteration of the filter. The reason is mainly reflected in the estimation of the number of targets, and the fundamental reason is that the two filters are essentially different in the implementation process.

**Figure 7:**
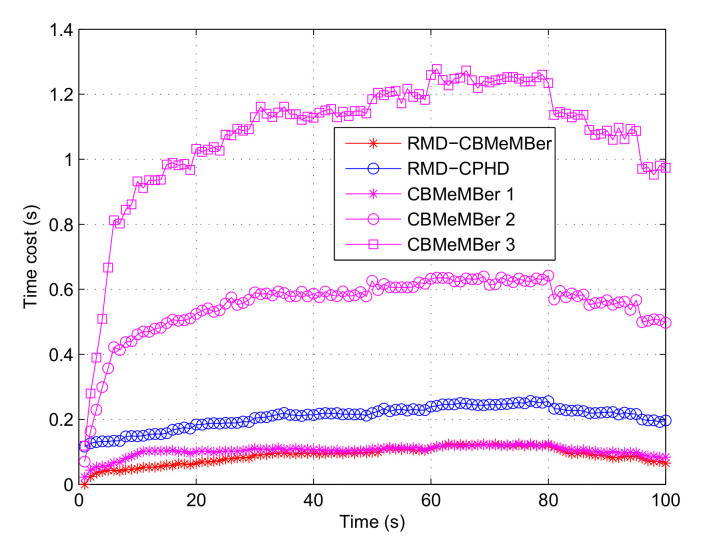
Statistics of time cost by using different algorithms (100 MC).

Figure 7 is a statistical graph of the time cost by employing different algorithms. Combining Figure 2, Figure 3, Figure 4, Figure 5, Figure 6 and Figure 7, it can be seen that the RMD-CBMeMBer filter proposed in this paper requires less time (200 particles in each newborn BC) to obtain the ideal multi-target state estimation result.

## 6. Conclusions

This paper proposes a new RMD-CBMeMBer filter in order to solve the multi-target tracking problem in the case of unknown background and lack of target prior information. The following conclusions are obtained through numerical experiments: First, the RMD-CBMeMBer filter proposes a correlation function, and uses the function to realize adaptive generation of BCs under the unknown background, which solves the problem of the lack of target prior information. Second, when the clutter rate and detection probability are unknown, the RMD-CBMeMBer filter achieves an effective estimation of the detection probability and average clutter rate of the target by extending the target state and modeling the detection probability and clutter density separately. In summary, the RMD-CBMeMBer filter proposed in this paper can achieve more efficient simultaneous tracking of multiple targets and maintain relatively stable tracking performance in the case of the target detection probability, clutter rate, and prior location information of the target being unknown.

## Figures and Tables

**Table 1:** Dynamic information of the true target.

	Initial State ($\left[m,m,m/s,m/s,\mathrm{rad}/s\right]$)	Time Interval ($\left[s,s\right]$)
1	$\left[800,1300,-8,-10,\pi /720\right]$	$\left[1,100\right]$
2	$\left[-550,1000,\;20,\;3,-\pi /270\right]$	$\left[5,95\right]$
3	$\left[-1500,250,\;11,\;10,-\pi /180\right]$	$\left[8,95\right]$
4	$\left[-1200,250,33,\;0,\;0\right]$	$\left[10,86\right]$
5	$\left[350,750,15,\;5,\pi /360\right]$	$\left[15,80\right]$
6	$\left[-250,1000,-12,-12,\pi /180\right]$	$\left[20,100\right]$
7	$\left[1000,800,\;0,-10,\pi /360\right]$	$\left[25,100\right]$
8	$\left[250,750,-35,-6,-\pi /360\right]$	$\left[30,80\right]$
9	$\left[1000,1000,-50,\;0,-\pi /360\right]$	$\left[50,100\right]$
10	$\left[250,750,-40,\;20,\pi /360\right]$	$\left[60,100\right]$
